# Systematic review of patient-oriented interventions to reduce unnecessary use of antibiotics for upper respiratory tract infections

**DOI:** 10.1186/s13643-020-01359-w

**Published:** 2020-05-08

**Authors:** Sameh Mortazhejri, Patrick Jiho Hong, Ashley M. Yu, Brian Younho Hong, Dawn Stacey, R. Sacha Bhatia, Jeremy M. Grimshaw

**Affiliations:** 1 School of Epidemiology and Public Health, University of Ottawa, Ottawa, Canada; 2 Clinical Epidemiology Program, Ottawa Hospital Research Institute, Ottawa, Canada; 3 Department of Anesthesiology and Pain Medicine, University of Toronto, Toronto, Canada; 4 Department of Family Medicine, McMaster University, Hamilton, Canada; 5 Division of Plastic and Reconstructive Surgery, University of Toronto, Toronto, Canada; 6 Faculty of Health Sciences, University of Ottawa, Ottawa, Canada; 7 Institute for Health System Solutions and Virtual Care, Women’s College Hospital, Toronto, Canada; 8 Insitute of Health Policy, Management and Evaluation, University of Toronto, Toronto, Canada; 9 Faculty of Medicine, University of Ottawa, Ottawa, Canada

**Keywords:** Antibiotic, Delayed prescription, Meta-analysis, Patient-oriented intervention, Systematic review, Upper respiratory tract infections

## Abstract

**Background:**

Antibiotics are prescribed frequently for upper respiratory tract infections (URTIs) even though most URTIs do not require antibiotics. This over-prescription contributes to antibiotic resistance which is a major health problem globally. As physicians’ prescribing behaviour is influenced by patients’ expectations, there may be some opportunities to reduce antibiotic prescribing using patient-oriented interventions. We aimed to identify these interventions and to understand which ones are more effective in reducing unnecessary use of antibiotics for URTIs.

**Methods:**

We conducted a systematic review by searching the Cochrane Central Register of Controlled Trials (CENTRAL), MEDLINE (OVID), EMBASE (OVID), CINAHL, and the Web of Science. We included English language randomized controlled trials (RCTs), quasi-RCTs, controlled before and after studies, and interrupted time series (ITS) studies. Two authors screened the abstract/titles and full texts, extracted data, and assessed study risk of bias. Where pooling was appropriate, a meta-analysis was performed by using a random-effects model. Where pooling of the data was not possible, a narrative synthesis of results was conducted.

**Results:**

We included 13 studies (one ITS, one cluster RCTs, and eleven RCTs). All interventions could be classified into two major categories: delayed prescriptions (seven studies) and patient/public information and education interventions (six studies). Our meta-analysis of delayed prescription studies observed significant reductions in the use of antibiotics for URTIs (OR = 0.09, CI 0.03 to 0.23; six studies). A subgroup analysis showed that prescriptions that were given at a later time and prescriptions that were given at the index consultation had similar effects. The studies in the patient/public information and education group varied according to their methods of delivery. Since only one or two studies were included for each method, we could not make a definite conclusion on their effectiveness. In general, booklets or pamphlets demonstrated promising effects on antibiotic prescription, if discussed by a practitioner.

**Conclusions:**

Patient-oriented interventions (especially delayed prescriptions) may be effective in reducing antibiotic prescription for URTIs. Further research is needed to investigate the costs and feasibility of implementing these interventions as part of routine clinical practice.

**Systematic review registration:**

PROSPERO CRD42016048007.

## Background

One third of primary care visits are because of infectious diseases, and half of these visits are for respiratory tract infections [1]. Antibiotics are prescribed frequently for upper respiratory tract infections (URTIs) by family physicians in Canada [2] and other parts of the world [3–8] despite the fact that most URTIs are viral, self-limiting, and commonly resolve without further complications [9]. Recent systematic reviews reveal small or no benefit from the antibiotics for most URTIs [10–12].

Excessive and inappropriate prescriptions of antibiotics can lead to antibiotic resistance [13, 14]. Antibiotic resistance is associated with high economic burden to the society and increases the length of hospital stay and mortality of inpatients [15, 16]. Prescription of unnecessary antibiotics can expose more people to the risk of adverse drug effects and drug interactions [17–19].

As patients are the end consumers of antibiotics, there may be opportunities for engaging patients’ attempts to reduce inappropriate antibiotic use. Studies have shown that physicians’ prescribing behaviour can be affected by patients’ (real or perceived) expectations about medications [20–22]. Interventions that influence patients’ behaviours, attitudes, and/or knowledge may be helpful in decreasing the unnecessary use of antibiotics.

We conducted a systematic review of all current literature to identify the interventions directed at patients to reduce unnecessary use of antibiotics and to better understand the ones that are more effective.

## Method

We conducted a systematic review of existing studies. Prior to undertaking the review, we registered the protocol in PROSPERO (ID = CRD42016048007). The Preferred Reporting Items for Systematic reviews and Meta-Analyses (PRISMA) checklist [23] was applied as a writing and reporting guideline (see Additional file 1).

### Criteria for considering studies for this review (Table 1)

#### Types of studies

We included randomized controlled trials (RCTs), quasi-RCTs, controlled before and after studies (CBA), and interrupted time series (ITS) studies. We used Cochrane Consumers and Communication Review Group (CC&CRG) eligibility guidance for CBA and ITS studies [24].

**Table 1:** Criteria for considering studies for the review

Study characteristics	Include	Exclude
**Participants**	Members of general public or patients of all age groups with upper respiratory tract infections (such as sinusitis, pharyngitis, sore throat, otitis media, common cold, and acute cough) who seek treatment in any general practice setting.	Patients with lower respiratory tract infections (LRTIs) and those with chronic lung conditions (such as chronic obstructive pulmonary disease (COPD)) will be excluded.
**Interventions**	Any intervention that is directed to patients, parents of patients (when the patients are children), public, or healthy individuals to reduce unnecessary use of antibiotics for URTIs in the primary care setting.	Interventions that are directed to healthcare providers or clinical staff will be excluded. The interventions that target patients indirectly (the primary and main effect of the intervention are directed to healthcare providers and patients benefit secondarily from that effect) will be excluded. Patient decision aids
**Comparisons**	These comparisons will be included: • Interventions directed at patients/public versus no intervention. • Interventions directed at patients/public versus standard or usual care. • One form of intervention directed at patients/public versus another.	Other comparisons will be excluded.
**Outcomes**	Primary outcomes: • Prescription or use of antibiotics for URTIs in the primary care setting. Secondary outcomes: • Public/patients’ satisfaction with the treatment/consultation. • Public/patients’ beliefs that antibiotics are effective for URTIs. • Re-consultation for the same illness in the next 2 weeks.	Studies that do not report the primary outcome will be excluded.
**Study designs**	Randomized controlled trials (RCTs) Quasi-RCTs (a trial in which randomization is attempted but subject to potential manipulation, such as allocating participants by day of the week, date or birth, or sequence of entry into trial). CBA (controlled before and after) studies are included if: • There are at least two intervention sites and two control sites; • The timing of the periods for study for the control and intervention groups is comparable (that is, the pre- and post- intervention periods of measurement for the control and intervention groups should be the same); • The intervention and control groups are comparable on key characteristics. ITS (interrupted time series) studies will be included if: • The intervention occurred at a clearly defined point in time, and this was specified by the researchers; • There were at least three data points before and three data points after the intervention was introduced.	CBA and ITS will not be included if they do not meet the mentioned criteria. Other kinds of studies (e.g. observational, reviews) will be excluded.
**Language**	English studies will be included.	Studies of other languages will be excluded.

#### Types of participants

Participants were members of the general public or patients of all age groups with URTIs (e.g. sinusitis, pharyngitis, sore throat, otitis media, common cold, and acute cough) who sought treatment in any general practice setting. Patients with lower respiratory tract infections (LRTIs) and those with chronic lung conditions (such as chronic obstructive pulmonary disease (COPD)) were excluded.

### Types of interventions

The types of interventions were as follows: patient-oriented interventions (i.e. directed to patients, parents of patients (in the case of paediatric patients), members of the general public) to reduce unnecessary use of antibiotics for URTIs in the primary care setting. Interventions that were directed to healthcare providers or clinical staff were excluded.

### Types of comparisons

We compared [24] interventions directed at patients/public versus no intervention, interventions directed at patients/public versus standard or usual care, and one form of intervention directed at patients/public versus another.

### Types of outcome measures

Our primary outcome was prescription of antibiotics by physicians or use of antibiotics by patients for URTIs in the primary care setting. Studies that did not report the primary outcome were excluded. Secondary outcomes were public/patients’ satisfaction with the treatment or consultation, public/patients’ beliefs about the effectiveness of antibiotics for URTIs, and re-consultation with a physician for the same illness.

### Type of language

We only included studies that were published in English.

## Search methods for identification of studies

We developed a search strategy combining terms for patient-oriented interventions, antibiotics, respiratory tract infections, and primary care settings in MEDLINE (OVID) (see Additional file 2) with the help of a librarian and adapted it to search other databases. Both MeSH terms and keywords were applied. The following databases were searched:

- MEDLINE (OVID): 1946 to 2016 November 11
- EMBASE (OVID): 1974 to 2016 November 11
- Cochrane Central Register of Controlled Trials (CENTRAL) including the Cochrane Effective Practice and Organisation of Care (EPOC) and Cochrane Consumer Network (CCNet): inception to 2016 October 23
- CINAHL: 1981 to 2016 October 24
- Web of Science: 1900 to 2016 October 28

World Health Organization (WHO), International Clinical Trials Registry Platform (ICTRP), and www.clinicaltrials.gov were also searched to detect completed and ongoing trials.

## Data collection and analysis

### Selection of studies

**Fig. 1:**
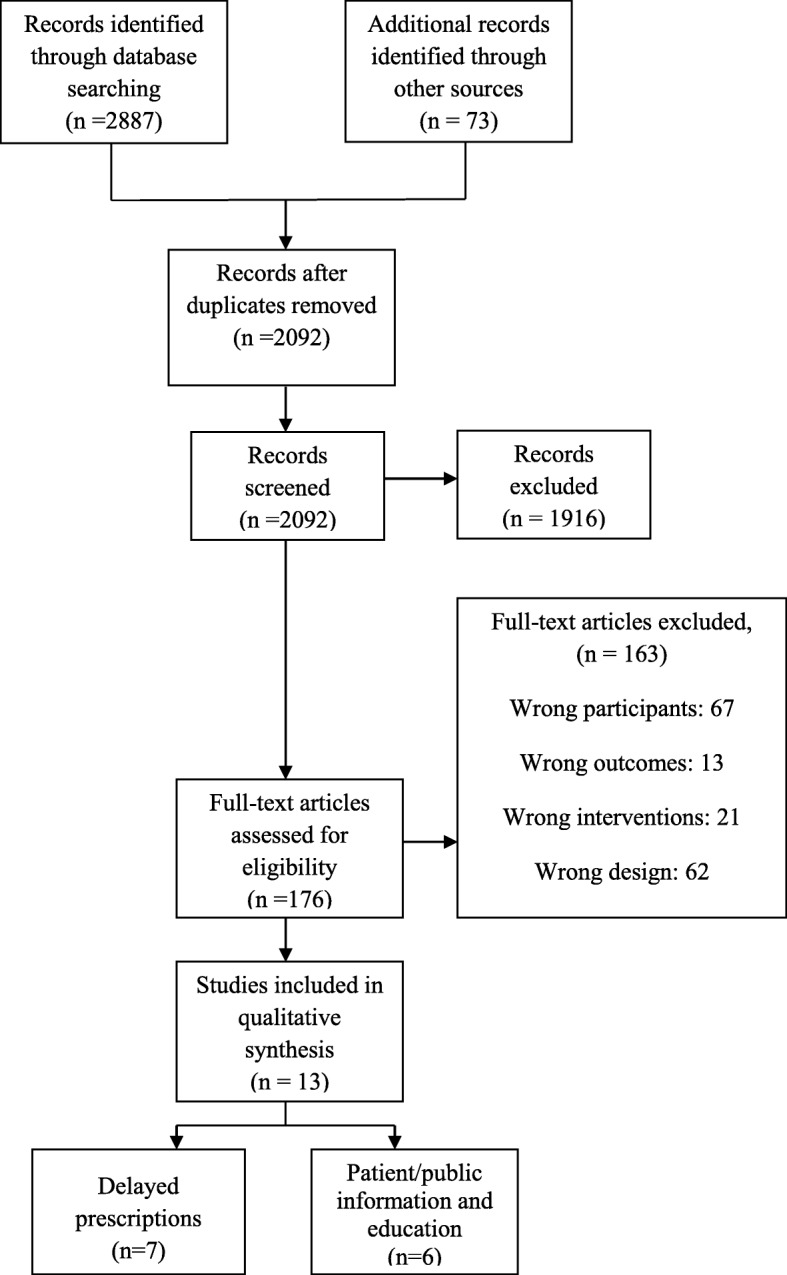
Study flow diagram

The study selection process was based on the PRISMA flow diagram [23] (Fig. 1). Two review authors (SM and either AY, PH, or BH) independently screened all titles and abstracts to determine study eligibility. They screened the full texts independently and in duplicate following title and abstract screening. In cases of uncertainty, complete manuscripts were reviewed, discussed, and resolved through consensus or discussion with a third party (JG). All potentially relevant papers that were excluded at this stage were listed as excluded studies. The references of all included studies were screened to identify potentially relevant articles.

### Data extraction and management

Two review authors (SM and either AY, PH, or BH) independently extracted the data from included studies. Any discrepancies were resolved by discussion until consensus was reached. The data extraction form was developed based upon EPOC and Cochrane Consumers and Communication group (CCCG) guides [25, 26] and included the key characteristics (methods, participants, interventions, outcomes, results) of included studies.

Study authors were contacted for additional information whenever there was an ambiguity in methods or data.

### Risk of bias assessments

Two reviewers (SM and either AY, PH, or BH) independently assessed the risk of bias of included studies in accordance with the risk of bias assessment guide of EPOC [27]. The items that were considered for RCT and CBA studies included allocation sequence generation, allocation concealment, similarity of baseline outcome measurements, similarity of the baseline characteristics, addressing of incomplete outcome data, prevention of knowledge of the allocated interventions during the study, contamination, and selective outcome reporting. The items that were examined for ITS studies included intervention independency of other changes, pre-specification of the shape of the intervention effect, likelihood of the intervention affecting data collection, prevention of knowledge of the allocated interventions during the study, addressing incomplete outcome data, and selective outcome reporting.

### Measures of intervention effect

For dichotomous outcomes, data were analysed based on the number of events and the number of people assessed in the intervention and comparison groups. These data were used to calculate the odds ratio (OR) and 95% confidence interval (CI). For continuous measures, data were used to calculate mean differences (the absolute difference between the mean value in two groups) (MD) and 95% CI. For studies with more than one intervention group, data was split in the control group to provide multiple two-arm comparisons [28].

### Dealing with common methodological issues

#### Unit of analysis issues in cluster-allocated studies

Studies that allocate at the cluster level (cluster RCTs and CBA studies) and analyse at the cluster member level need to account for the clustered nature of the data during analysis. Failure to do this results in over-precise results (i.e. small *P* values) but not biased effect sizes [28]. Therefore, we checked whether the analysis of such studies had taken account of clustering. If not, we made a note in the limitations of the study and only reported the pointed effect estimate without mentioning the CI or *P* values.

#### Inappropriate analysis of ITS studies

We evaluated the method of analysis in ITS studies to check if they compared time trends before and after the intervention. If an appropriate analysis was not provided in the original paper, we re-analysed the data by extracting data points from the graphs [29]. We performed a segmented time series regression model to estimate the effect of the intervention taking into account time trends and autocorrelation.

#### Dealing with missing data

There were no missing data, but there was some incompatibility between data presented in tables and data presented in the text of the articles, where we contacted the authors to seek clarification. Where we did not hear back from the authors within 6 months, raw numbers were used in the analysis.

### Data synthesis

We categorized the studies according to the type of their interventions. Afterwards, we inspected the studies within categories of similar interventions to see if there was sufficient similarity to consider pooling. If pooling was possible, Review Manager 5 software [30] was used to meta-analyse the studies using a random-effects model. Where pooling the data using meta-analysis was not possible, a narrative synthesis of results was conducted. The similarities and differences between the findings of studies were investigated, as well as the exploration of patterns in the data [31]. We conducted subgroup analysis based on subtypes of the intervention. We presumed different subtypes of the intervention would influence the effect size and explain heterogeneity.

#### Assessment of heterogeneity

We assessed heterogeneity among studies by visual inspection of forest plots and by examining the *I*^2^ statistic. *I*^2^ statistic describes the percentage of variation across studies that is due to heterogeneity rather than chance [32].

#### Assessment of reporting biases

We planned to assess the risk of publication bias by using a funnel plot. However, because less than 10 studies were suitable for pooling, we could not use this method to assess the publication bias [33].

#### Sensitivity analysis

We conducted sensitivity analysis by investigating the effect of omitting studies that were outliers compared to other studies in the forest plot.

## Results

### Description of studies

#### Results of the search

The literature search resulted in 2092 unique records after de-duplication (Fig. 1). After title and abstract screening, 176 studies were included for full-text evaluation. Following full-text screening, a total of 13 studies were selected for inclusion in the review. See Additional file 3 for details of excluded studies (see Additional file 3).

#### Included studies

Full details of the included studies are available in the “characteristics of included studies” table (Table 2 and Additional file 4). We included 11 RCTs, one cluster RCT, and one study that we re-analysed as ITS. Most studies were conducted in Europe (eight studies) with a few other studies from the USA (one study), Canada (one study), Asia (two studies), and New Zealand (one study). Studies were completed between 1997 and 2016.

**Table 2:** Summary of characteristics of included studies

Study	Methods	Participants	Interventions	Primary outcome
**Delayed prescription**				
Arroll et al. [34]	RCT, 2 arms. Settings: New Zealand, one family practice (15 physicians)	Patients presenting with the common cold. Number of participants: 129	Delayed antibiotic prescription	Antibiotic use
Little et al. [35]	RCT, 3 arms. Settings: UK, general practices	Patients with sore throat. Number of participants: 716	Delayed prescription of antibiotics, no antibiotics	Antibiotic use
Little et al. [36]	RCT, 2 arms. Settings: UK, general practices	Children with acute otalgia. Number of participants: 315	Delayed prescription of antibiotics	Use and collection of antibiotic prescriptions
Little et al. [37]	RCT, 5 arms. Settings: UK, primary care setting	Patients with a respiratory tract infection. Number of participants: 556	Delayed patient-led, post-dated prescription, delayed-collection, delayed-re-contact, no prescription	Symptom severity
Pshetizky et al. [38]	RCT, 2 arms. Settings: Israel, primary care setting	Parents of children with acute otitis media. Number of participants: 81	Delayed prescription of antibiotics	Antibiotic use
Poza Abad et al. [39]	RCT, 4 arms. Settings: Spain, primary care setting	Patients with uncomplicated respiratory infections. Number of participants: 398	Delayed patient-led, delayed collection, no antibiotic	Duration and severity of symptoms
Worrall et al. [40]	RCT, 2 arms. Settings: Canada, primary care setting	Adult patients with acute URTIs. Number of participants: 149	Post-dated delayed antibiotic prescription	Filling the prescription by the patients
**Patient/public information and education**				
Alexandrino et al. [41]	RCT, 2 arms. Settings: Portugal, 10 private daycare centres	Caregivers. Number of participants: 177	Health education session (HES)	Impact of HES on the indicators of individual health and health care utilization
Francis et al. [42]	Cluster RCT, 61 clusters, 2 arms. Settings: UK, general practices	Children with a respiratory tract infection. Number of participants: 558	Interactive booklet	The proportion of children who attended a face-to-face consultation about the same illness during the 2-week follow-up period
Lambert et al. [43]	ITS. Settings: UK, a single geographical population in the North East of England	People of the community	Mass media education	Prescribing rates for all microbial agents
Lee et al. [44]	RCT, 2 arms. Settings: Singapore, primary care setting	Patients presenting RTI symptoms. Number of participants: 914	Education through pamphlets and counselling scripts	Antibiotic prescription
Little et al. [45]	RCT, 2 arms. Settings: UK, primary care setting	Adult patients. Number of participants: 2923	Interactive website	General practitioner consultation
Taylor et al. [46]	RCT, 2 arms. Settings: USA, primary care setting	Healthy children. Number of participants: 499	Parental education through pamphlets and videos	Number of diagnoses of otitis media and sinusitis per study child, number of visits per child for which antibiotics (oral or intramuscular) were prescribed

#### Interventions

All interventions were classified into two major categories: (1) delayed prescriptions [34–40], and (2) patient/public information and education [41–46]. Two studies compared different types of delayed prescription [37, 39]. Patient education was provided through pamphlets, booklets, or videotapes in three studies [42, 44, 46] through educational sessions in one study [41] and via online educational program in one study [45]. One study [43] used mass media as an educational tool.

#### Outcomes

Included studies reported a wide range of outcomes; the most common were use of antibiotics, prescription of antibiotics, collection or filling of prescriptions by patients, satisfaction with the treatment, satisfaction with the consultation, patients’ beliefs about the effectiveness of antibiotics, and re-consultation with a physician for the same or similar episodes of URTIs (Table 2).

#### Funding sources

Nine studies reported being funded by the government or research foundation funds; two studies reported no funding; two studies did not declare the sources of their funding.

### Risk of bias in included studies

#### RCT/cluster RCT

**Fig. 2:**
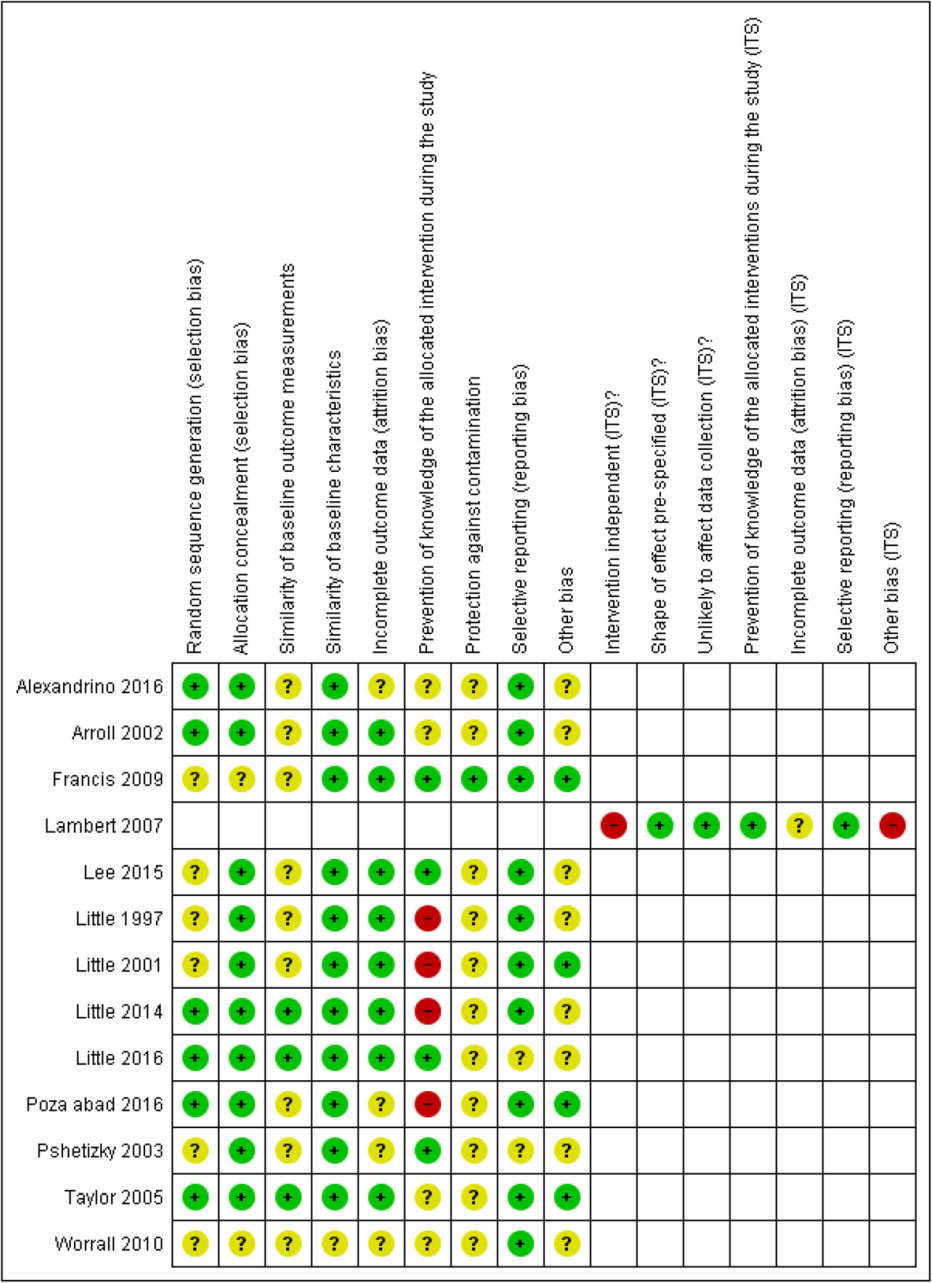
Risk of bias summary

Regarding random sequence generation, allocation concealment, and similarity of baseline characteristics, all studies were of low or unclear risk of bias (Fig. 2). All studies were judged to be low or unclear risk for addressing incomplete outcome data, protection against contamination, and selective outcome reporting. Four studies [35–37, 39] were assigned high risk of bias regarding prevention of knowledge of allocated interventions because the participants were not blinded to the interventions.

#### ITS

The main source of bias in the ITS study [43] was that the intervention was not independent of other changes, and the authors mentioned that they were unable to control or document the complementary interventions. Also, the method of analysis of the data did not take into account the time trends before and after the interventions.

### Effects of interventions

#### Primary outcome

##### 1) Delayed prescription (seven studies)

Four studies compared immediate prescription of antibiotics with delayed prescription in a two-arm RCT design [34, 36, 38, 40]. Three studies [35, 37, 39] used multi-arm RCTs to evaluate different types of delayed prescriptions. The types of delayed prescriptions evaluated were:

Delayed patient-led: the delayed prescription was given to the patients at the time of the initial visit, and patients were given instructions to fill the prescription after a given time period (2 to 3 days, depending on the study) [34, 37–39].

Post-dated prescription: the delayed prescription was given at the time of the visit; however, it was post-dated [37, 40].

Delayed collection: the delayed prescription was not provided to patients at the time of the visit, but rather was lodged at the practice’s reception or pharmacy, and patients were invited to collect or fill their prescription if their symptoms had not improved or worsened after a few days (2 to 7 days, depending on the study) [35–37, 39].

Delayed re-contact: patients were asked to contact or phone and leave a message to request antibiotics [37].

A few studies [35, 37, 39] included a group of no antibiotic prescription along with other intervention groups. We did not use the data from this group in our analysis.

##### 1.1) Analysis of data comparing delayed prescription with immediate prescription

**Fig. 3:**
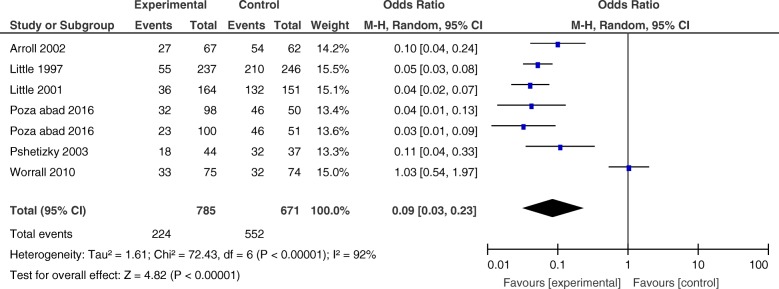
Forest plot-comparing antibiotic use between intervention and control groups in the delayed prescription group

We performed a meta-analysis of six RCT studies involving a total of 1788 participants that compared delayed prescription [34–36, 38–40] with an immediate prescription control group. The study by Little et al. could not be included because there was no immediate prescription control group within this study [37]. Most studies reported antibiotic use as one of their outcomes while one study [40] only reported the filling of the prescriptions by patients. We assumed that filling the prescription from the pharmacy will lead to antibiotic use, so data from all these studies were pooled. We used an intention-to-treat (ITT) approach to analyse the data by using the original number of participants that were randomized to intervention and control groups in each study. Overall, the participants in the delayed prescription group were less likely than participants in the immediate prescription group to use antibiotics (OR = 0.09, CI 0.03 to 0.23). There was considerable heterogeneity in the estimate of effect size among studies (*I*^2^ = 92%) (Fig. 3); we performed subgroup analysis to investigate the heterogeneity.

##### 1.1.a) Exploring the heterogeneity by subgroup analysis according to the time of prescription delivery

To explore the source of heterogeneity, we divided the studies into (1) studies in which the prescription was given at the time of the visit with some instructions to wait for a few days before filling it (delayed patient-led and post-dated prescription) [34, 38–40] and (2) studies in which the prescription was not given at the time of the visit, and patients were asked to return to collect the prescription after a few days (delayed collection) [35, 36, 39].

**Fig. 4:**
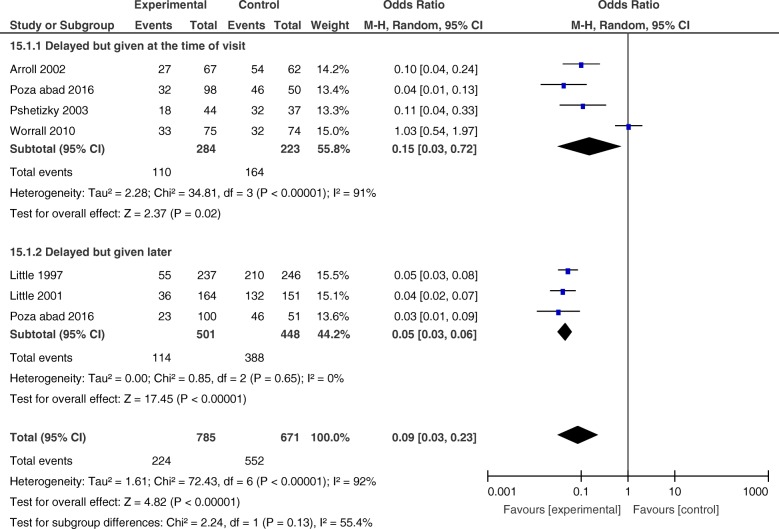
Forest plot-comparing antibiotic use between intervention and control groups, sub-grouped analysis

The OR of antibiotic use for delayed patient-led or post-dated was 0.15 (CI 0.03–0.72) with heterogeneity (*I*^2^) of 91%. The OR of antibiotic use for delayed collection was 0.05 (CI 0.03 to 0.06) with no heterogeneity (Fig. 4).

##### 1.1.b) Sensitivity analysis of data comparing delayed prescription with immediate prescription

**Fig. 5:**
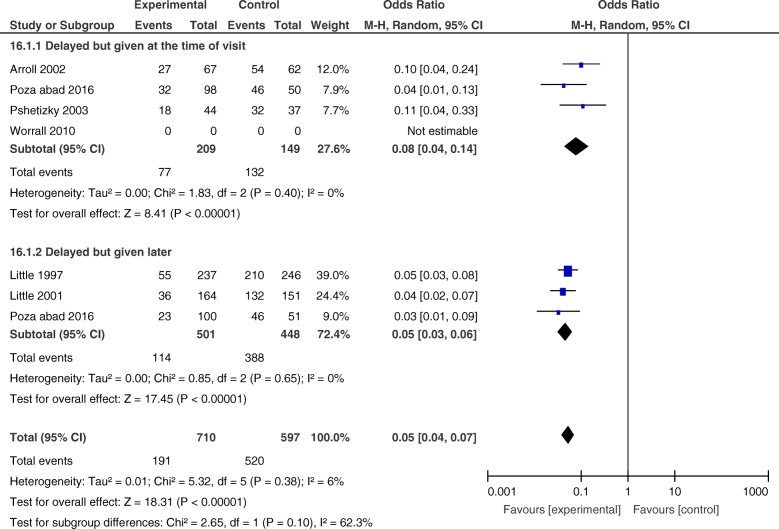
Forest plot-comparing antibiotic use between intervention and control groups after deleting Worrall et al. study

After sub-grouping the studies, there was still a considerable amount of heterogeneity in studies of delayed patient-led or post-dated prescriptions. Worrall et al. was the only study that showed almost no difference in antibiotic use between the intervention and control groups (OR = 1.03, CI 0.54 to 1.97) (Fig. 4) [40]. In this study, patients in both the intervention and control groups were informed about the self-limiting nature of the illness and were asked to use the prescription (post-dated prescription in the intervention group) if symptoms had not improved or had worsened after 2 days. Furthermore, patients in the intervention group were able to fill the prescriptions earlier than the assigned date. These issues could cause similar antibiotic use in the intervention and control groups. To determine the robustness of the results, we re-analysed the data omitting the Worrall et al. study (Fig. 5) and observed similar ORs for delayed patient-led or post-dated prescriptions and delayed collection (OR = 0.08, CI 0.04 to 0.14, and OR = 0.05, CI 0.03 to 0.06, respectively). Furthermore, omitting the Worrall et al. study resulted in an overall OR of 0.05 (CI 0.04 to 0.07) and total heterogeneity (*I*^2^) of 6% (Fig. 5).

##### 1.2) Analysis of data comparing different types of delayed prescription

Little et al. studied five intervention groups: delayed patient-led, delayed collection, post-dated prescription, delayed re-contact, and no antibiotic prescription. Their study showed no significant difference in antibiotic use between these groups. This analysis did not compare antibiotic use between delayed prescription methods and immediate prescription, but only demonstrated that different methods of delayed prescription appear to have similar effects on antibiotic use (likelihood ratio test = 4.96, *P* value = 0.292) [37].

##### 2) Patient/public information and education methods

Studies in this group differed based on their educational material, methods of providing the education and reported outcomes. Thus, it was not possible to perform a meta-analysis.

##### 2.1) Pamphlets/booklets/videotapes (three studies)

Parents of healthy children younger than 24 months old seen in the offices of participating clinics in the Taylor et al. study received “your child and antibiotics” pamphlet (see Additional file 4), as well as a video that emphasized the main points of the pamphlet. Additional copies of the pamphlets were mailed to the parents at 6 weeks and 6 months after enrolment. The authors reported no significant differences in the total number of prescriptions for antibiotics per patient in the intervention and control groups during the 12-month observation period (MD = − 0.3, *P* value = 0.23) [46].

The pamphlets that were used in the Lee et al. study provided education on the causes of URTIs and the role of antibiotics and were designed to address the major misconceptions about URTIs that were recognized in the previous local studies. The researcher verbally educated participants by using the pamphlets. Participants were patients aged 21 years and above, presenting with URTI symptoms at participating clinics. The results of the study demonstrated no significant difference in antibiotic prescription between the intervention and control groups (OR = 1.20, CI 0.84 to 1.72) [44].

In a cluster RCT, Francis et al. used booklets on RTIs in children (6 months to 14 years) consulting with a respiratory tract infection and their parents. The booklets included information on the effectiveness of antibiotic treatment, potential adverse effects from antibiotics, other treatment suggestions, and symptoms that should prompt re-consultation and were used within the consultations by physicians, as well as a resource to be taken home by participants. The authors mentioned proper adjustments for the effect of clustering. The OR of antibiotic prescription at the index consultation was 0.29 in the favour of intervention (CI 0.14 to 0.60). The authors also reported the OR taking antibiotics within the first 2 weeks as 0.35 (CI 0.18 to 0.66) [42].

##### 2.2) Educational sessions plus booklet (one study)

One study used educational sessions to inform parents or legal tutors of paediatric patients (children under 3 years old in daycare centres). Alexandrino et al. used the “Health Education Sessions” (HES) (see Additional file 4) of mean duration of 90 min delivered by a respiratory physiotherapist in small groups of 10 to 15 caregivers (parents or legal tutors). The sessions included information on prevention of acute respiratory infections (ARIs), signs and symptoms of ARIs, signs of worsening, medications, and nasal clearance techniques. The participants were also provided with a booklet with a summary of the information at the end of the sessions. The results showed less antibiotic use in the intervention group compared to the control group (OR = 0.33, CI 0.12 to 0.90) [41].

##### 2.3) Interactive online educational program (one study)

Little et al. [45] provided the participants (a random selection of adults in the computerized practice registers from 35 practices) with an access to an interactive website for 20 weeks. The website delivered tailored advice on visiting/not visiting a physician and methods of self-management for URTIs. The results of the study showed that antibiotic prescription did not differ significantly between the intervention and control groups in the first 6 months after the intervention (RR = 1.02, CI 0.82 to 1.43) or after longer follow-ups (12 months) (RR = 1.00, CI 0.74 to 1.33) [45].

##### 2.4) Mass media (one study)

Lambert et al. used a retrospective CBA design to evaluate the effects of two sequential mass media campaigns on the prescription of antibiotics. The campaigns consisted of a short cartoon strip about the effects of antibiotics and self-care for managing self-limited health problems. This was accompanied by leaflets, posters, and TV (was added in the second intervention), radio, and local newspaper coverage. The authors used a repeated measures analysis of variance to analyse monthly prescribing data in the intervention and control populations. Their analysis did not control for baseline differences or secular trends, nor for multiple testing. Given the limited conclusions which could be drawn from their analysis, Plot Digitizer software was used to extract data for the intervention series over observation period and data were re-analysed using an ITS approach. Our analysis did not incorporate the control population but allowed the effect of the intervention to be assessed in the intervention population, while controlling for the pre-intervention level and secular trend. The results were adjusted for autocorrelation. Regarding the prescription rate, after the first intervention, the change in slope was 0.39 (CI − 1.87 to 2.65) and the change in level was − 8.94 (CI − 23.31 to 5.42). After the second intervention, the change in slope was calculated as − 2.11 (CI − 5.75 to 1.54) and the change in level as − 1.40 (CI − 17.40 to 14.60) [43].

#### Secondary outcomes

Secondary outcome measures of interest that were reported included patients’ satisfaction with the treatment or consultation, patients’ beliefs on the effectiveness of antibiotics for URTIs, and re-consultation. A summary of these results is provided in Table 3.

**Table 3:** The effect of interventions on patients’ satisfaction, beliefs and re-consultation

Study	Patients’ satisfaction^**1**^	Antibiotic Beliefs^**2**^	Re-consultation^**3**^
**Delayed prescription**			
Arroll et al. [34] (delayed vs. immediate)	Intervention 96% Control 94% OR = 1.47 (CI 0.32 to 6.85)	Intervention 76% Control 76% OR = 1.02 (CI 0.45 to 2.28)	Intervention 73% Control 65% OR = 1.50 (CI 0.71 to 3.17)
Little et al. [35] (delayed vs. immediate)	Intervention 93% Control 96% OR = 0.61 (CI 0.25 to 1.49)	Intervention 60% Control 87% OR = 0.22 (CI 0.13 to 0.36)	Intervention 57% Control 79% OR = 0.35 (CI 0.22 to 0.55)
Little et al. [36] (delayed vs. immediate)	Intervention 77% Control 91% OR = 0.32 (CI 0.16 to 0.65)	Intervention 46% Control 76% OR = 0.24 (CI 0.14 to 0.40)	Intervention 63% Control 83% OR = 0.35 (CI 0.20 to 0.62)
Little et al. [37]^**4**^ (different variants of delayed prescriptions vs. no antibiotic)	Delayed re-contact 74% Post-dated 80% Delayed collection 88% Delayed patient-led 89% Likelihood ratio test *X*^2^ = 2.38 (*P* value = 0.67)	Delayed re-contact 74% Post-dated 73% Delayed collection 72% Delayed patient-led 66% Likelihood ratio test *X*^2^ = 1.62 (*P* value = 0.80)	Delayed re-contact 18% Post-dated 10% Delayed collection 14% Delayed patient-led 14% Likelihood ratio test *X*^2^ = 2.97 (*P* value = 0.56)
Poza Abad^5^ et al. [39] (delayed collection vs. immediate and delayed patient-led vs. immediate)	*P* value^6^ = 0.14	Delayed collection 84.4% Immediate 91.8% OR = 0.37 (CI 0.14 to 0.99) Delayed patient-led 81% Immediate 91.8% OR = 0.37 (CI 0.14 to 0.98)	Delayed collection 69.1% Immediate 85.7% OR = 0.62 (CI 0.19 to 2.06) Delayed patient-led 69.0% Immediate 85.7% OR = 0.35(CI 0.1 to 1.30)
**Patient/public information and intervention**			
Francis et al. [42] (interactive booklet vs. control)	Intervention 90.2% Control 93.5% OR = 0.64 (CI 0.33 to 1.22)		Intervention 55.3% Control 76.4% OR = 0.34 (CI 0.20 to 0.57)
Little et al. [45] (online educational program vs. control)			Intervention 19.3% Control 19.3% OR = 0.93 (CI 0.73 to 1.16)

^1^The outcome was reported as “satisfaction with the consultation” in all studies except for Little [36] study, in which it was reported as “satisfaction with the treatment approach”. OR > 1 means more satisfaction in the intervention group compared to the control group. OR < 1 means less satisfaction in the intervention group compared to the control group

^2^The outcome was belief in the effectiveness of antibiotics for URTIs. OR < 1 means less belief in the effectiveness of antibiotics in the intervention group compared to the control group. OR > 1 means more belief in the effectiveness of antibiotics in the intervention group compared to the control group

^3^Re-consultation was reported only in Little [37] (within 1 month after the consultation) and Little [45] (within 1 year after enrolment) studies; all other studies reported the intention to re-consult in future. OR < 1 means less re-consultation in the intervention group compared to the control group. OR > 1 means more re-consult in the intervention group compared to the control group

^4^All intervention groups (different variants of delayed prescriptions) were compared to “no antibiotic”

^5^The *P* values are reported to compare the difference between delayed collection, delayed patient-led, immediate prescription, and no prescription

^6^No more data were available

##### Patients’ satisfaction with the treatment or consultation

Five studies reported patients’ satisfaction with the consultation, and one study reported the patients’ satisfaction with the treatment. Some of these studies reported the number of participants who were “very satisfied”, some combined the “very satisfied” and “moderately satisfied” groups together, and some studies did not mention any further details.

In the delayed prescription group, two studies from the UK reported less satisfaction in the intervention group compared to the control group, though the results were statistically significant in only one study [35, 36]. One study from New Zealand reported higher satisfaction (though this was not statistically significant) in the intervention group [34]. In the Little et al. study from the UK, there was no significant difference in satisfaction between different variants of delayed prescription [37]. Poza Abad et al. from Spain reported no significant difference in satisfaction between delayed collection, delayed patient-led, and immediate prescription groups [39].

In the patient/public information and education group, only one study (from the UK) which used booklets as intervention measured satisfaction. It showed less satisfaction in the intervention group; however, it was not statistically significant [42].

##### Patients’ beliefs on the effectiveness of antibiotics for URTIs

Four studies compared participants’ beliefs on the effectiveness of antibiotics between the delayed prescription group and immediate prescription group. Two studies from the UK and one study from Spain showed significant results in the favour of intervention [35, 36, 39]. One study from New Zealand reported no difference between control and intervention groups [34]. In the Little et al. study there was no significant difference in patients’ beliefs between different variants of delayed prescription [37]. No studies in the information and education group measured this outcome.

##### Re-consultation

Five studies in the delayed prescription group evaluated patients’ willingness to re-consult for similar illnesses in the near future or re-consultation rate. Two studies from the UK and one study from Spain [35, 36, 39] reported less intention to re-consult in the intervention groups; however, the results were statistically significant only in two studies [35, 36]. One study from New Zealand reported greater intention to re-consult in the intervention group though this was not statistically significant [34]. In the Little et al. study (from the UK), there was no significant difference in re-consultation between different variants of delayed prescription [37].

Two studies in the patient/public information and education group (from the UK) (one applied booklets, the other applied online educational program) reported less re-consultation in the intervention group, while the results were statistically significant in only one study [42, 45].

## Discussion

### Summary of main results

This systematic review synthesized evaluations of patient-oriented interventions to reduce unnecessary use of antibiotics for URTIs. The 13 studies that were included in our review focused on either delayed prescription of antibiotics or information and education materials as their interventions. Our meta-analysis revealed that almost all studies with delayed prescription significantly reduced use of antibiotics for URTIs. Our subgroup analysis showed that the prescriptions that were given at a later time and the prescriptions that were given at the index consultation had similar effects in reducing antibiotic use in patients.

The effect of interventions in the information and education group varied highly among different types of educational materials. The results suggested that providing information/education via online educational program or mass media (each evaluated in one study) might not have a significant effect on antibiotic prescription; however, one study [41] that used educational sessions plus booklets disclosed promising effects on reducing antibiotic use to those who attended. Furthermore, applying booklets, pamphlets, or videotapes demonstrated inconsistent results on antibiotic prescription; it seems that when the intervention was provided by a physician rather than a researcher and was discussed verbally in a face-to-face visit [42], it led to better results.

Changes in patients’ satisfaction, beliefs on the effectiveness of antibiotics, and re-consultation were only reported in a few studies. In the delayed prescription group, one study from New Zealand [34] reported higher satisfaction and more re-consultation in the intervention group (non-significant). However, the other studies in this group (from Spain and the UK) [35, 36, 39] showed the opposite results. The difference between the results of the study from New Zealand and other studies (Europe) might be explained in part by the location of studies. Differences in cultural or socioeconomic backgrounds of participants in different settings could have affected the results.

### Strengths and limitations of the study

We believe that one of the major strengths of this review is the comprehensive literature search in various databases; however, our study has a number of limitations:

We limited our search to English language studies which may have caused missing some studies in this field. We identified some studies where the educational interventions were directed at both patients and healthcare providers. We excluded these studies as we were interested in the effects of just targeting patients. We also excluded the studies that promoted shared decision making or communication between the patients and healthcare providers. However, we included the studies with delayed prescription, because it is the patient who decides to collect or fill the prescription. A few studies in our review included LRTIs in addition to URTIs [37, 39]; since the major focus of these studies was URTIs, we did not exclude them.

Our systematic review was dependent on the results provided in the included studies and therefore was influenced by the ways they reported their outcomes.

Some studies lacked clear data on the effect size and only provided *P* values. Secondary outcomes were measured and reported in multiple ways. Patients’ satisfaction and beliefs on the effectiveness of antibiotics for URTIs were examined using diverse questionnaires and scales. Re-consultation was defined with various times to follow-up, and some studies had evaluated patients’ intentions to re-consult in the future and not the actual re-consultation rate. All these differences in measuring and reporting the outcomes or lack of enough data made pooling the data inappropriate.

There were also two issues regarding the use of antibiotics. First, antibiotic use was measured by relying on patients’ reports in most of the studies. This may have introduced social desirability bias which could have distorted the estimates of antibiotic use. Second, some studies reported antibiotic prescription instead of antibiotic use. It is not clear how many of these prescriptions resulted in actual use of antibiotics.

### Agreements and disagreements with other studies

Other systematic reviews have evaluated the effect of different patient-oriented interventions on antibiotic use [47–53]. However, most of them have not evaluated the quality of included studies or have focused on only specific kind of interventions (e.g. delayed prescriptions). Some of them have only reported the effect of interventions on antibiotic use in children [48, 52].

In general, most reviews shared similar results. Consistent with our results, Thoolen et al., Spurling et al., Andrews et al., Arnold et al., and McDonagh et al. concluded that delayed prescriptions resulted in a significant decrease in antibiotic use [48–51, 53].

Similar to our results, there was not a consensus among reviews on the effectiveness of educational methods on reducing antibiotic use. One review reported no or small benefit from printed educational materials [52]. O’Sullivan et al. examined the effect of written information for patients and concluded that providing written information to parents of children can lead to a decrease in antibiotic use. However, they included only two studies in their review, and it is difficult to make firm conclusions about the effectiveness of all written information for patients based on these sparse data [47]. McDonagh et al. concluded that clinic-based educational materials for parents (e.g. posters, pamphlets, interactive videos) reduce overall prescription of antibiotics. Some of the differences between the results of this study and other studies could be explained by differences in their inclusion criteria. A broader range of designs including observational studies were included in this study. Consistent with our results, McDonagh et al. agreed that public educational campaigns are not effective in reducing antibiotic use [53].

We only included studies that had been conducted in primary care settings; however, some of the studies in emergency department share the same results as our study [54, 55].

### Implications for practice

The results of this study are consistent with other studies [48–51, 53] that delayed prescription of antibiotics reduces the antibiotic use for URTIs. This strategy can be adopted by healthcare providers and policy makers. To implement this strategy, we may need educational strategies for physicians to explain to the patients when and how the prescriptions can be accessed.

The effect of interventions that only focused on patients’ or public education varied according to the type of materials used and the way they were applied. We found the interventions that were delivered face to face or through a session and were discussed verbally by the physicians showed promising effects on decreasing antibiotic use or prescription. This highlights the role of active education versus passive methods and the importance of physicians’ role to explain the contents of educational materials to patients.

### Implications for research

The effectiveness of different patient-oriented interventions on reducing antibiotic use has been evaluated in multiple studies. Conducting more studies of this kind (especially about the delayed prescriptions) in similar settings does not seem to provide any new insights in understanding their effectiveness. However, we have limited data on patients’ satisfaction with these interventions. In our review, the studies from the UK and New Zealand showed different direction of effect for satisfaction, suggesting that there may be cultural or contextual factors that modify the intervention acceptability. Research is needed to investigate the factors that affect intervention acceptability and therefore patients’ satisfaction among different settings.

There may also be some opportunities in combining different components of effective interventions to design new multifaceted interventions (e.g. mixing delayed prescriptions with pamphlets/booklets). Further research is needed to identify and evaluate the most effective combinations.

Better reporting of interventions’ details (who delivered the interventions, the settings in which they were delivered, how often they were delivered) would make it easier to compare the interventions or to adopt them. Most studies in our review also lacked the description of co-interventions or assessment of fidelity.

Some studies in our review reported antibiotic prescription as their outcome. However, not all patients actually use their prescriptions. On the other hand, the studies that reported antibiotic use instead of antibiotic prescription relied on patients’ self reports which may introduce desirability bias. It is important to choose a common outcome measure to allow us to measure the real antibiotic use by patients.

Finally, qualitative research can help to realize why some interventions are more effective in some settings and less effective in others. These methods can also be beneficiary in understanding patients’ concerns with the treatments or consultations in order to achieve a higher satisfaction.

## Conclusion

Our study focused on addressing patients to decrease the unnecessary use of antibiotics for URTIs. Patient-oriented interventions have been studied in two major categories: delayed antibiotic prescription and patient/public information and education materials. There is evidence that delayed prescription of antibiotics reduces antibiotic use by patients. The effects of educational intervention varied among different educational methods and materials. It seems that providing education through sessions or pamphlets/booklets (especially if delivered by a healthcare provider and discussed verbally) may decrease antibiotic use or prescription.

## Supplementary information

**Additional file 1.** The PRISMA checklist.

**Additional file 2.** Search strategy.

**Additional file 3.** Excluded studies and reasons for exclusion.

**Additional file 4.** Characteristics of included studies.

## Data Availability

All data generated or analysed during this study are included in this published article (and its supplementary information files).

## Abbreviations

AOM: Acute otitis media
ARI: Acute respiratory infections
CBA: Controlled before after
CC&CRG: Cochrane Consumers and Communication Review Group
CCCG: Cochrane Consumers and Communication Group
CCNet: Cochrane Consumers Network
CENTRAL: Cochrane Central Register of Controlled Trials
CI: Confidence interval
COPD: Chronic obstructive pulmonary disease
EPOC: Effective Practice and Organization of Care
GP: General practitioner
ICTRP: International Clinical Trials Registry Platform
ITS: Interrupted time series
ITT: Intention To Treat
LRTI: Lower respiratory tract infection
MD: Mean difference
OR: Odds ratio
PRISMA: Preferred items for systematic reviews and meta-analyses
RCT: Randomized controlled trial
URTI: Upper respiratory tract infections
WHO: World Health Organization
