# Supplementary material for: Systematic review of patient-oriented interventions to reduce unnecessary use of antibiotics for upper respiratory tract infections
Source: Syst Rev. 2020 May 8;9:106. doi: 10.1186/s13643-020-01359-w (PMC7210679; doi:10.1186/s13643-020-01359-w)
Supplement: Supplementary file 2 — Additional file 2. Search strategy. [file 13643_2020_1359_MOESM2_ESM.docx]

**Additional file 2**

**Search strategy**

| 1. exp Patient Education as Topic/ |
| --- |
| 2. exp Consumer Health Information/ |
| 3. exp Pamphlets/ |
| 4. exp Decision Support Techniques/ |
| 5. exp Health Promotion/ |
| 6. exp Computer-Assisted Instruction/ |
| 7. motivational interviewing/ or distance counselling/ |
| 8. counselling/ or directive counselling/ |
| 9. exp Information Services/ |
| 10. exp Mass Media/ |
| 11. exp Social Media/ |
| 12. self care/ or exp self administration/ or exp self medication/ |
| 13. health education/ or exp consumer health information/ or exp health fairs/ or exp teach-back communication/ |
| 14. exp Teaching Materials/ |
| 15. exp Patient Participation/ |
| 16. exp Decision Making/ |
| 17. exp social support/ |
| 18. exp Consumer Participation/ |
| 19. exp Cooperative Behaviour/ |
| 20. exp Behaviour Therapy/ |
| 21. exp Problem Solving/ |
| 22. exp Postal Service/ |
| 23. exp "Delivery of Health Care"/ |
| 24. exp "Cost Sharing"/ |
| 25. exp Insurance, Health, Reimbursement/ or exp Reimbursement, Incentive/ |
| 26. exp Motivation/ |
| 27. (Delay* adj2 prescrip*).ti,ab. |
| 28. (Health adj2 Promot*).ti,ab. |
| 29. (Computer* adj2 assist* adj2 education?).ti,ab. |
| 30. (Motivat* adj2 Interview*).ti,ab. |
| 31. Counsel*.ti,ab. |
| 32. (Inform* adj2 Service*).ti,ab. |
| 33. mass media.ti,ab. |
| 34. social media.ti,ab. |
| 35. self care.ti,ab. |
| 36. (health adj2 educat*).ti,ab. |
| 37. (teaching adj2 material?).ti,ab. |
| 38. (decision? adj2 mak*).ti,ab. |
| 39. decision support techniques/ |
| 40. decision aid*.ti,ab. |
| 41. (patient? adj2 participat*).ti,ab. |
| 42. (patient? adj2 involv*).ti,ab. |
| 43. (patient? adj2 support?).ti,ab. |
| 44. (social adj2 support?).ti,ab. |
| 45. (patient? adj2 educat*).ti,ab. |
| 46. (facilitat* adj2 communicat*).ti,ab. |
| 47. (health adj2 inform*).ti,ab. |
| 48. pamphlet?.ti,ab. |
| 49. booklet?.ti,ab. |
| 50. (educat* adj3 video?).ti,ab. |
| 51. (educat* adj3 book?).ti,ab. |
| 52. brochure?.ti,ab. |
| 53. (Computer* adj2 interact*).ti,ab. |
| 54. (health* adj2 behavior?).ti,ab. |
| 55. (health* adj2 behaviour?).ti,ab. |
| 56. (consumer? adj2 particip*).ti,ab. |
| 57. (support* adj2 behavior?).ti,ab. |
| 58. (support* adj2 behaviour?).ti,ab. |
| 59. (cooperat* adj2 behavior?).ti,ab. |
| 60. (cooperat* adj2 behaviour?).ti,ab. |
| 61. (inform* adj2 material?).ti,ab. |
| 62. (educat* adj3 video?).ti,ab. |
| 63. (behavior adj2 therapy).ti,ab. |
| 64. (behaviour adj2 therapy).ti,ab. |
| 65. (problem adj2 solving).ti,ab. |
| 66. Group programme?.ti,ab. |
| 67. action plan?.ti,ab. |
| 68. post* service?.ti,ab. |
| 69. Mass mailing?.ti,ab. |
| 70. (mail* adj2 distribut*).ti,ab. |
| 71. (mail? adj2 order*).ti,ab. |
| 72. (Financial adj2 intervention?).ti,ab. |
| 73. (Financial adj2 incentive?).ti,ab. |
| 74. Harm reduc*.ti,ab. |
| 75. (cost? adj2 shar*).ti,ab. |
| 76. co-payment?.ti,ab. |
| 77. copayment?.ti,ab. |
| 78. reimburs*.ti,ab. |
| 79. (health adj2 campaign?).ti,ab. |
| 80. (wellness adj2 campaign?).ti,ab. |
| 81. (consumer? adj2 involv*).ti,ab. |
| 82. 1 or 2 or 3 or 4 or 5 or 6 or 7 or 8 or 9 or 10 or 11 or 12 or 13 or 14 or 15 or 16 or 17 or 18 or 19 or 20 or 21 or 22 or 23 or 24 or 25 or 26 or 27 or 28 or 29 or 30 or 31 or 32 or 33 or 34 or 35 or 36 or 37 or 38 or 39 or 40 or 41 or 42 or 43 or 44 or 45 or 46 or 47 or 48 or 49 or 50 or 51 or 52 or 53 or 54 or 55 or 56 or 57 or 58 or 59 or 60 or 61 or 62 or 63 or 64 or 65 or 66 or 67 or 68 or 69 or 70 or 71 or 72 or 73 or 74 or 75 or 76 or 77 or 78 or 79 or 80 or 81 |
| 83. exp Respiratory Tract Infections/ |
| 84. exp Sinusitis/ |
| 85. sinusitis.ti,ab. |
| 86. respiratory tract infection?.ti,ab. |
| 87. (respiratory adj2 infection?).ti,ab. |
| 88. exp Pharyngitis/ |
| 89. pharyngitis.ti,ab. |
| 90. sore throat?.ti,ab. |
| 91. exp Laryngitis/ |
| 92. laryngitis.ti,ab. |
| 93. exp Tonsillitis/ |
| 94. Tonsil?itis.ti,ab. |
| 95. exp Otitis Media/ |
| 96. otitis media.ti,ab. |
| 97. (inflammation? adj2 middle ear).ti,ab. |
| 98. exp Common Cold/ |
| 99. common cold?.ti,ab. |
| 100. chest cold?.ti,ab. |
| 101. coryza?.ti,ab. |
| 102. catarrh?.ti,ab. |
| 103. exp Rhinitis/ |
| 104. Rhinitis.ti,ab. |
| 105. rhinosinusitis.ti,ab. |
| 106. exp Epiglottitis/ |
| 107. epiglotitis.ti,ab. |
| 108. Epiglottitis.ti,ab. |
| 109. exp Tracheitis/ |
| 110. Tracheitis.ti,ab. |
| 111. exp Influenza, Human/ |
| 112. influenza?.ti,ab. |
| 113. flu?.ti,ab. |
| 114. flue?.ti,ab. |
| 115. grippe?.ti,ab. |
| 116. pharyngalgia?.ti,ab. |
| 117. pharyngeal pain?.ti,ab. |
| 118. (pharyn* adj2 infection?).ti,ab. |
| 119. (pharyn* adj2 inflammation?).ti,ab. |
| 120. throat infection?.ti,ab. |
| 121. strep* throat.ti,ab. |
| 122. throat pain?.ti,ab. |
| 123. throat ache?.ti,ab. |
| 124. 83 or 84 or 85 or 86 or 87 or 88 or 89 or 90 or 91 or 92 or 93 or 94 or 95 or 96 or 97 or 98 or 99 or 100 or 101 or 102 or 103 or 104 or 105 or 106 or 107 or 108 or 109 or 110 or 111 or 112 or 113 or 114 or 115 or 116 or 117 or 118 or 119 or 120 or 121 or 122 or 123 |
| 125. 82 and 124 |
| 126. exp Anti-Bacterial Agents/ |
| 127. antibiotic?.ti,ab. |
| 128. antibacterial?.ti,ab. |
| 129. anti?bacterial?.ti,ab. |
| 130. antibiotic?.ti,ab. |
| 131. antimycobacterial?.ti,ab. |
| 132. anti?mycobacterial?.ti,ab. |
| 133. bacteriocid*.ti,ab. |
| 134. antibacter*.ti,ab. |
| 135. exp Penicillins/ |
| 136. penicillin?.ti,ab. |
| 137. exp Amoxicillin-Potassium Clavulanate Combination/ or exp Amoxicillin/ |
| 138. amoxicillin.ti,ab. |
| 139. coamoxiclav*.ti,ab. |
| 140. amoxi-Clavulanate.ti,ab. |
| 141. amoxiClavulanate.ti,ab. |
| 142. Co-Amoxiclav*.ti,ab. |
| 143. exp Cephalosporins/ |
| 144. Cephalosporin*.ti,ab. |
| 145. exp Ceftriaxone/ |
| 146. ceftriaxone.ti,ab. |
| 147. exp Cefixime/ |
| 148. cefixime.ti,ab. |
| 149. Azithromycin/ |
| 150. azitrocin.ti,ab. |
| 151. Azit?romycin.ti,ab. |
| 152. Cefotaxime/ |
| 153. cefotaxime.ti,ab. |
| 154. exp Ampicillin/ |
| 155. ampicillin?.ti,ab. |
| 156. Doxycycline/ |
| 157. doxycycline.ti,ab. |
| 158. Cephalexin/ |
| 159. cephalexin.ti,ab. |
| 160. Erythromycin/ |
| 161. erythromycin.ti,ab. |
| 162. betalactam?.ti,ab. |
| 163. beta?lactam?.ti,ab. |
| 164. anti?biotic?.ti,ab. |
| 165. 126 or 127 or 128 or 129 or 130 or 131 or 132 or 133 or 134 or 135 or 136 or 137 or 138 or 139 or 140 or 141 or 142 or 143 or 144 or 145 or 146 or 147 or 148 or 149 or 150 or 151 or 152 or 153 or 154 or 155 or 156 or 157 or 158 or 159 or 160 or 161 or 162 or 163 or 164 |
| 166. 125 and 165 |
| 167. exp Primary Health Care/ |
| 168. primary care.ti,ab. |
| 169. primary health care.ti,ab. |
| 170. physicians/ or general practitioners/ or physicians, family/ or physicians, primary care/ |
| 171. family physician?.ti,ab. |
| 172. family doctor?.ti,ab. |
| 173. general doctor?.ti,ab. |
| 174. general physician?.ti,ab. |
| 175. general practitioner?.ti,ab. |
| 176. family practitioner?.ti,ab. |
| 177. exp Family Practice/ |
| 178. family practice.ti,ab. |
| 179. general practice.ti,ab. |
| 180. General Practice/ |
| 181. first-line care?.ti,ab. |
| 182. first line care?.ti,ab. |
| 183. general medical practice.ti,ab. |
| 184. GP?.ti,ab. |
| 185. 167 or 168 or 169 or 170 or 171 or 172 or 173 or 174 or 175 or 176 or 177 or 178 or 179 or 180 or 181 or 182 or 183 or 184 |
| 186. 166 and 185 |
