# Supplementary material for: Systematic review of patient-oriented interventions to reduce unnecessary use of antibiotics for upper respiratory tract infections
Source: Syst Rev. 2020 May 8;9:106. doi: 10.1186/s13643-020-01359-w (PMC7210679; doi:10.1186/s13643-020-01359-w)
Supplement: Supplementary file 3 — Additional file 3. Excluded studies and reasons for exclusion. [file 13643_2020_1359_MOESM3_ESM.docx]

**Additional file 3**

**Excluded studies and reasons for exclusion**

| **Study** | **Year** | **Reason for exclusion** |
| --- | --- | --- |
| Belongia et al. [1] | 2005 | ITS: insufficient number of observations. |
| Cebotarenco et al. [2] | 2008 | CBA: Only one site. |
| Dowel et al. [3] | 2001 | The primary outcome was reported only in the intervention group. |
| Flottorp et al. [4] | 2002 | Multifaceted intervention: not possible to disentangle the effects of patient-oriented interventions. |
| Grover et al. [5] | 2013 | Before after study without a control group. |
| LeBlanc et al. [6] | 2011 | Multifaceted intervention: not possible to disentangle the effects of patient-oriented interventions. |
| Llor et al. [7] | 2014 | Multifaceted intervention: not possible to disentangle the effects of patient-oriented interventions. |
| Mainous et al. [8] | 2000 | CBA: Only one site. |
| Malmvall et al. [9] | 2007 | Multifaceted intervention: not possible to disentangle the effects of patient-oriented interventions. |
| McNulty et al. [10] | 2010 | CBA: Only one site. |
| Meeker et al. [11] | 2014 | Multifaceted intervention: not possible to disentangle the effects of patient-oriented interventions. |
| Plachouras et al. [12] | 2014 | Multifaceted intervention: not possible to disentangle the effects of patient-oriented interventions. |
| Rubin et al. [13] | 2005 | CBA: Only one site. |
| Sabuncu et al. [14] | 2009 | ITS: insufficient number of observations. |
| Welschen et al. [15] | 2004 | Multifaceted intervention: not possible to disentangle the effects of patient-oriented interventions. |

**References:**

1. Belongia EA, Knobloch MJ, Kieke BA, Davis JP, Janette C, Besser RE. Impact of statewide program to promote appropriate antimicrobial drug use. Emerg Infect Dis. 2005 Jun;11(6):912–20.

2. Cebotarenco N, Bush PJ. Reducing antibiotics for colds and flu: a student-taught program. Health Educ Res. 2008 Feb;23(1):146–57.

3. [Dowell J](https://www.ncbi.nlm.nih.gov/pubmed/?term=Dowell%20J%5BAuthor%5D&cauthor=true&cauthor_uid=11255901), [Pitkethly M](https://www.ncbi.nlm.nih.gov/pubmed/?term=Pitkethly%20M%5BAuthor%5D&cauthor=true&cauthor_uid=11255901), [Bain J](https://www.ncbi.nlm.nih.gov/pubmed/?term=Bain%20J%5BAuthor%5D&cauthor=true&cauthor_uid=11255901), [Martin S](https://www.ncbi.nlm.nih.gov/pubmed/?term=Martin%20S%5BAuthor%5D&cauthor=true&cauthor_uid=11255901). A randomised controlled trial of delayed antibiotic prescribing as a strategy for managing uncomplicated respiratory tract infection in primary care. Br J Gen Pract. 2001;51(464):200–5.

4. [Flottorp S](https://www.ncbi.nlm.nih.gov/pubmed/?term=Flottorp%20S%5BAuthor%5D&cauthor=true&cauthor_uid=12183309), [Oxman AD](https://www.ncbi.nlm.nih.gov/pubmed/?term=Oxman%20AD%5BAuthor%5D&cauthor=true&cauthor_uid=12183309), [Håvelsrud K](https://www.ncbi.nlm.nih.gov/pubmed/?term=H%C3%A5velsrud%20K%5BAuthor%5D&cauthor=true&cauthor_uid=12183309), [Treweek S](https://www.ncbi.nlm.nih.gov/pubmed/?term=Treweek%20S%5BAuthor%5D&cauthor=true&cauthor_uid=12183309), [Herrin J](https://www.ncbi.nlm.nih.gov/pubmed/?term=Herrin%20J%5BAuthor%5D&cauthor=true&cauthor_uid=12183309). Cluster randomised controlled trial of tailored interventions to improve the management of urinary tract infections in women and sore throat. Br Med J. 2002;325(7360):367–70.

5. Grover ML, Nordrum JT, Mookadam M, Engle RL, Moats CC, Noble BN. Addressing antibiotic use for acute respiratory tract infections in an academic family medicine practice. Am J Med Qual. 2013;28(6):485–91.

6. Leblanc A, Legare F, Labrecque M, Godin G, Thivierge R, Laurier C, et al. Feasibility of a randomised trial of a continuing medical education program in shared decision-making on the use of antibiotics for acute respiratory infections in primary care: the DECISION+ pilot trial. Implement Sci. 2011;6:5.

7. [Llor C](https://www.ncbi.nlm.nih.gov/pubmed/?term=Llor%20C%5BAuthor%5D&cauthor=true&cauthor_uid=24768657), [Cots JM](https://www.ncbi.nlm.nih.gov/pubmed/?term=Cots%20JM%5BAuthor%5D&cauthor=true&cauthor_uid=24768657), [Hernández S](https://www.ncbi.nlm.nih.gov/pubmed/?term=Hern%C3%A1ndez%20S%5BAuthor%5D&cauthor=true&cauthor_uid=24768657), [Ortega J](https://www.ncbi.nlm.nih.gov/pubmed/?term=Ortega%20J%5BAuthor%5D&cauthor=true&cauthor_uid=24768657), [Arranz J](https://www.ncbi.nlm.nih.gov/pubmed/?term=Arranz%20J%5BAuthor%5D&cauthor=true&cauthor_uid=24768657), [Monedero MJ](https://www.ncbi.nlm.nih.gov/pubmed/?term=Monedero%20MJ%5BAuthor%5D&cauthor=true&cauthor_uid=24768657) et al. Effectiveness of two types of intervention on antibiotic prescribing in respiratory tract infections in Primary Care in Spain. Happy Audit Study. [Aten Primaria.](https://www.ncbi.nlm.nih.gov/pubmed/24768657) 2014 Nov;46(9):492-500.

8. [Mainous AG 3rd](https://www.ncbi.nlm.nih.gov/pubmed/?term=Mainous%20AG%203rd%5BAuthor%5D&cauthor=true&cauthor_uid=10645510), [Hueston WJ](https://www.ncbi.nlm.nih.gov/pubmed/?term=Hueston%20WJ%5BAuthor%5D&cauthor=true&cauthor_uid=10645510), [Love MM](https://www.ncbi.nlm.nih.gov/pubmed/?term=Love%20MM%5BAuthor%5D&cauthor=true&cauthor_uid=10645510), [Evans ME](https://www.ncbi.nlm.nih.gov/pubmed/?term=Evans%20ME%5BAuthor%5D&cauthor=true&cauthor_uid=10645510), [Finger R](https://www.ncbi.nlm.nih.gov/pubmed/?term=Finger%20R%5BAuthor%5D&cauthor=true&cauthor_uid=10645510). An evaluation of statewide strategies to reduce antibiotic overuse. [Fam Med.](https://www.ncbi.nlm.nih.gov/pubmed/?term=An+Evaluation+of+Statewide+Strategies+to+Reduce+Antibiotic+Overuse) 2000 Jan;32(1):22-9.

9. [Malmvall BE](https://www.ncbi.nlm.nih.gov/pubmed/?term=Malmvall%20BE%5BAuthor%5D&cauthor=true&cauthor_uid=17235252), [Mölstad S](https://www.ncbi.nlm.nih.gov/pubmed/?term=M%C3%B6lstad%20S%5BAuthor%5D&cauthor=true&cauthor_uid=17235252), [Darelid J](https://www.ncbi.nlm.nih.gov/pubmed/?term=Darelid%20J%5BAuthor%5D&cauthor=true&cauthor_uid=17235252), [Hiselius A](https://www.ncbi.nlm.nih.gov/pubmed/?term=Hiselius%20A%5BAuthor%5D&cauthor=true&cauthor_uid=17235252), [Larsson L](https://www.ncbi.nlm.nih.gov/pubmed/?term=Larsson%20L%5BAuthor%5D&cauthor=true&cauthor_uid=17235252), [Swanberg J](https://www.ncbi.nlm.nih.gov/pubmed/?term=Swanberg%20J%5BAuthor%5D&cauthor=true&cauthor_uid=17235252), et al. Reduction of antibiotic sales and sustained low incidence of bacterial resistance: report on a broad approach during 10 years to implement evidence-based indications for antibiotic prescribing in Jonkoping County, Sweden. Qual Manag Health Care. 2007 Jan;16(1):60–7.

10. McNulty CAM, Nichols T, Boyle PJ, Woodhead M, Davey P. The English antibiotic awareness campaigns: did they change the public’s knowledge of and attitudes to antibiotic use? J Antimicrob Chemother. 2010 Jul;65(7):1526–33.

11. [Meeker D](https://www.ncbi.nlm.nih.gov/pubmed/?term=Meeker%20D%5BAuthor%5D&cauthor=true&cauthor_uid=24474434), [Knight TK](https://www.ncbi.nlm.nih.gov/pubmed/?term=Knight%20TK%5BAuthor%5D&cauthor=true&cauthor_uid=24474434), [Friedberg MW](https://www.ncbi.nlm.nih.gov/pubmed/?term=Friedberg%20MW%5BAuthor%5D&cauthor=true&cauthor_uid=24474434), [Linder JA](https://www.ncbi.nlm.nih.gov/pubmed/?term=Linder%20JA%5BAuthor%5D&cauthor=true&cauthor_uid=24474434), [Goldstein NJ](https://www.ncbi.nlm.nih.gov/pubmed/?term=Goldstein%20NJ%5BAuthor%5D&cauthor=true&cauthor_uid=24474434), [Fox CR](https://www.ncbi.nlm.nih.gov/pubmed/?term=Fox%20CR%5BAuthor%5D&cauthor=true&cauthor_uid=24474434) et al. Nudging guideline-concordant antibiotic prescribing: A randomized clinical trial. [JAMA Intern Med.](https://www.ncbi.nlm.nih.gov/pubmed/24474434) 2014 Mar;174(3):425-31.

12. [Plachouras D](https://www.ncbi.nlm.nih.gov/pubmed/?term=Plachouras%20D%5BAuthor%5D&cauthor=true&cauthor_uid=25149626), [Antoniadou A](https://www.ncbi.nlm.nih.gov/pubmed/?term=Antoniadou%20A%5BAuthor%5D&cauthor=true&cauthor_uid=25149626), [Giannitsioti E](https://www.ncbi.nlm.nih.gov/pubmed/?term=Giannitsioti%20E%5BAuthor%5D&cauthor=true&cauthor_uid=25149626), [Galani L](https://www.ncbi.nlm.nih.gov/pubmed/?term=Galani%20L%5BAuthor%5D&cauthor=true&cauthor_uid=25149626), [Katsarolis I](https://www.ncbi.nlm.nih.gov/pubmed/?term=Katsarolis%20I%5BAuthor%5D&cauthor=true&cauthor_uid=25149626), [Kavatha D](https://www.ncbi.nlm.nih.gov/pubmed/?term=Kavatha%20D%5BAuthor%5D&cauthor=true&cauthor_uid=25149626) et al. Promoting prudent use of antibiotics: the experience from a multifaceted regional campaign in Greece. BMC Public Health. 2014;14:866.

13. Rubin MA, Bateman K, Alder S, Donnelly S, Stoddard GJ, Samore MH. A Multifaceted Intervention to Improve Antimicrobial Prescribing for Upper Respiratory Tract Infections in a Small Rural Community. [Clin Infect Dis](https://www.ncbi.nlm.nih.gov/pubmed/15712077). 2005 Feb 15;40(4):546-53.

14. Sabuncu E, David J, Bernede-Bauduin C, Pepin S, Leroy M, Boelle PY, et al. Significant reduction of antibiotic use in the community after a nationwide campaign in France, 2002-2007. PLoS Med. 2009 Jun;6(6):e1000084.

15. Welschen I, Kuyvenhoven MM, Hoes AW, Verheij TJ. Effectiveness of a multiple intervention to reduce antibiotic prescribing for respiratory tract symptoms in primary care: randomised controlled trial. Br Med J. 2004 Aug;329(7463):431
