# Supplementary material for: Systematic review of patient-oriented interventions to reduce unnecessary use of antibiotics for upper respiratory tract infections
Source: Syst Rev. 2020 May 8;9:106. doi: 10.1186/s13643-020-01359-w (PMC7210679; doi:10.1186/s13643-020-01359-w)
Supplement: Supplementary file 4 — Additional file 4. Characteristics of included studies. [file 13643_2020_1359_MOESM4_ESM.docx]

**Additional file 4**

**Characteristics of included studies**

| **Alexandrino 2016** | |
| --- | --- |
| **Methods** | RCT, 2 arms  **Study setting**: Portugal, 10 private daycare centers. |
| **Participants** | Caregivers (parents or legal tutors) of children aged up to 3 years old.  **Number of participants**: 177 |
| **Interventions** | Health Education Session (HES) about respiratory infections on the indicators of individual health and health care utilization of daycare children. The HES had a mean duration of 1 hour and 30 minutes. It covered the following five domains: prevention of ARI, first signs and symptoms of ARI, worsening signs of ARI, medication and nasal clearance techniques.  The HES was conducted by a respiratory physiotherapist among small groups of 10–15 caregivers at the daycare centre. At the end of the HES, the participants received a small booklet with a summary of the information. |
| **Outcomes** | **Primary:**  Impact of HES on the indicators of individual health and health care utilization (signs of respiratory infections, respiratory infections diagnosed by the child’s doctor, health care utilization of medical consultations, emergency services and/or antibiotics, absenteeism, nasal clearance techniques).  **Secondary:**  Association between the caregivers’ use of nasal clearance techniques and respiratory infections.   - Antibiotic use was measured by filling a diary of records by participants. |
| **Notes** | **Funding:** none |

| **Arroll 2002** | |
| --- | --- |
| **Methods** | RCT, 2 arms  **Study setting**: New Zealand, one family practice (15 physicians) |
| **Participants** | Patients presenting with the common cold who requested antibiotics or whose physicians thought they wanted them. For young children, the parents indicated whether or not they wanted antibiotics.  **Number of participants**: 129 |
| **Interventions** | Delayed antibiotic prescription: the Intervention group was given a prescription for antibiotics with instructions to fill it after 3 days if symptoms failed to improve. |
| **Outcomes** | **Primary:**  Antibiotic use (taking at least one dose of antibiotic)  **Secondary:**  Symptom score, satisfaction, patient's intention to visit a physician for the next cold, patients' beliefs on the effectiveness of antibiotics.   - No details on measurement of antibiotic use. |
| **Notes** | **Funding:** Health Research Council |

| **Francis 2009** | |
| --- | --- |
| **Methods** | Cluster RCT, 61 clusters, 2 arms  **Study setting**: UK, general practices. |
| **Participants** | Children (6 months to 14 years) consulting with a respiratory tract infection (cough, cold, sore throat, earache for seven days or less) and their parents.  **Number of participants**: 558 |
| **Interventions** | Interactive booklet on respiratory tract infections. It consisted of eight pages on respiratory tract infections in children, designed to be used within the consultation and then provided to parents as a take home resource, to facilitate the use of certain communication skills, mainly exploring the parent’s main concerns, asking about their expectations, and discussing prognosis, treatment options, and any reasons that should prompt re-consultation. |
| **Outcomes** | **Primary:**  The proportion of children who attended a face-to-face consultation about the same illness during the two week follow-up period.  **Secondary:**  Antibiotic prescribing, antibiotic consumption, future consulting intentions, and parental satisfaction, reassurance, and enablement.   - Antibiotic use was measured by a telephone administered questionnaire with the child’s parent or guardian. |
| **Notes** | **Funding:** Medical Research Council and the Welsh Assembly Government in the form of a joint Health Services Fellowship for NF.  Funding for the development of the training website was from an educational grant from Pfizer UK. The South-East Wales Trials Unit was funded by the Welsh Office for Research and Development. This study was sponsored by Cardiff University. |

| **Lambert 2007** | |
| --- | --- |
| **Methods** | ITS  **Study setting**: UK, a single geographical population in the North East of England. |
| **Participants** | People of the community |
| **Interventions** | Mass media education. A regional mass media campaign used in two consecutive years. It was led by a cartoon character called ‘Moxy Malone’ who was developed specifically for this regional campaign. The character was given dialogue that included simple messages ‘facts’ and ‘fictions’ about the effects of antimicrobials, and about the availability of community pharmacists to support self-care for the management of usually self limiting health problems. These messages were drawn together under the title of ‘Antibiotics- tracking down the truth’, which appeared as a short cartoon strip, with supporting leaflets and posters. In addition, the campaign received editorial coverage from local newspapers, TV and radio. In both years, advertisements were run on local radio with posters on buses and local Metro system. Printed materials (leaflets and posters) were made available to GP surgeries throughout the target area. In the second phase, regional television advertising was added, with the same artwork and character.   - The timing of the campaigns was chosen to coincide with the annual peak in consultations for respiratory infections. |
| **Outcomes** | **Primary:**  Prescribing rates prescribing rates (items) for all microbial agents.  **Secondary:**  Analysing factors that had a possible effect on the prescribing of antibacterial drugs.   - The volumes prescribed were derived from the Prescription Pricing Authority database. |
| **Notes** | - The Moxy Malone campaign was not the only intervention being used to tackle inappropriate antimicrobial prescribing at this time. The undocumented co-interventions may have affected the results.   **Funding:** None. |

| **Lee 2017** | |
| --- | --- |
| **Methods** | RCT, 2 arms  **Study setting:** Singapore, primary care setting. |
| **Participants** | Patients aged 21 years and above, presenting with at least one of four URTI symptoms (runny nose, blocked nose, cough or sore throat) for seven days or less.  **Number of participants:** 914 |
| **Interventions** | Patients in the intervention arm were educated verbally and by using pamphlets on causes of URTIs and when antibiotics are appropriate to treat URTIs. The intervention pamphlets and counselling scripts specifically addressed key misconceptions identified in the previous study of URTI patients at local primary care clinics. |
| **Outcomes** | **Primary:**  The proportion of patients in each arm prescribed antibiotics.  **Secondary:**  Patients’ agreement on a 4-point scale to the following three statements: that the education had improved their understanding about causes of URTI, that they were worried about the side effects of antibiotics, and that antibiotics are not needed most of the time for URTI.   - Antibiotic prescription was measured by an interviewer-assisted post-consultation questionnaire. |
| **Notes** | **Funding:** Saw Swee Hock School of Public Health. |

| **Little 1997** | |
| --- | --- |
| **Methods** | RCT, 3 arms.  **Study setting:** UK, general practices. |
| **Participants** | Patients aged 4 years and over with sore throat and an abnormal physical sign in the throat;  **Number of participants**: 716 |
| **Interventions** | Intervention group 1: no antibiotics.  Intervention group 2: patients were offered antibiotics, but the patient could collect the prescription from the surgery if symptoms were not starting to settle within three days. |
| **Outcomes** | **Primary:**  Antibiotic use.  **Secondary:**  Duration of antibiotic use, resolution of symptoms, believe that antibiotics were effective, intention to consult in future episodes, satisfaction, time off school or work.   - Antibiotic use was measured by filling a daily diary by participants and a doctors’ documentation sheet. |
| **Notes** | **Funding:** This work was supported by Wessex NHS regional research and development funds. One of the authors was supported by the Wellcome Trust. |

| **Little 2001** | |
| --- | --- |
| **Methods** | RCT, 2 arms.  **Study setting:** UK, general practices. |
| **Participants** | Children aged 6 months to 10 years, who attended their doctor with acute otalgia and otoscopic evidence of acute inflammation of the ear drum (dullness or cloudiness with erythema, bulging, or perforation).  **Number of participants:** 315 |
| **Interventions** | Delayed treatment with antibiotics. Parents were asked to wait for 72 hours after seeing the doctor before considering using the prescription.  Parents were instructed that if their child still had substantial otalgia or fever after the 72 hours or was not starting to get better then they should come and collect the prescription for antibiotics, which was left at the practice's reception. |
| **Outcomes** | **Primary:**  Use and collection of antibiotic prescriptions.  **Secondary:**  Symptoms resolution and analgesia use, absence from school or nursery, satisfaction, belief that antibiotics are very effective, intention to consult doctor in future, side effects.   - Antibiotic use was measured by filling a daily diary by participants and a doctors’ documentation sheet. |
| **Notes** | **Funding**: NHS Research and Development (South West and South East Regions). One of the authors was supported by the Medical Research Council. |

| **Little 2014** | |
| --- | --- |
| **Methods** | RCT, 5 arms.  **Study setting:** UK, primary care setting. |
| **Participants** | Patients aged 3 years and over with a respiratory tract infection diagnosed by the health professional.  **Number of participants:** 556 |
| **Interventions** | Intervention groups:  1) Patient led: the patient was given antibiotics and asked to wait to use them. 2) Post-dating: the patient was given antibiotics, but post dated.  3) Collection: patients were given instructions to wait but could request antibiotics from front desk. 4) Re-contact/phone: patients were asked to contact/phone the surgery to leave message for doctor/nurse to request antibiotics and they were able to come to reception. 5) No offer of prescription but a clinical review for worsening symptoms. |
| **Outcomes** | **Primary:**  Symptom severity.  **Secondary:**  Any antibiotic use in the 14 days after recruitment, side effects, mean temperature, duration of symptoms, return with new or worsening symptoms or complications of intervention, belief in the effectiveness of antibiotics and satisfaction.   - Antibiotic use was measured by filling a diary by participants. |
| **Notes** | - A non-randomised group was added later in the study that received immediate prescription of antibiotics.   **Funding:** It was funded by the National Institute for Health Research under its Programme Grants for Applied Research programme. |

| **Little 2016** | |
| --- | --- |
| **Methods** | RCT, 2 arms.  **Study setting:** UK, primary care setting. |
| **Participants** | Adult patients (aged 18+ years).  **Number of participants:** 2923 |
| **Interventions** | Interactive website providing tailored advice. It was reinforced by email prompts and reminders to use the website; patients were given information about the natural history, self-care advice and advice about the use of over-the-counter medication.  Participants had access to the website for 20 weeks. On logging onto the website, users could select tailored advice on (1) whether and why they need/do not need to consult the GP and (2) how to self-care for RTIs. Patients selecting consultation advice completed questions about their symptoms and medical history, and were then presented with tailored advice recommending either self-management (for mild symptoms), for more severe symptoms phoning the ‘NHS Direct’ helpline, which provided nurse-led advice about the need to seek further medical help, or alternatively, seeking medical attention. Patients were given the opportunity to challenge this advice by selecting further in-depth information. |
| **Outcomes** | **Primary:**  General practitioner consultation.  **Secondary:**  The use of antibiotics, contacting NHS Direct for phone-based advice, the duration of symptoms, the number of days where work/normal activities were impaired, re-consultation, hospitalization.   - The use of antibiotics was documented as prescription of antibiotics, from patient records. |
| **Notes** | **Funding:** This study was funded by the National Institute for Health Research Programme Grants for Applied Research programme. |

| **Pshetizky 2003** | |
| --- | --- |
| **Methods** | RCT, 2 arms.  **Study setting**: Israel, primary care setting. |
| **Participants** | Parents of children aged 3 months to 4 years diagnosed with acute otitis media (AOM).  **Number of participants:** 81 |
| **Interventions** | The intervention group received a structured explanation and a prescription for antibiotics to be used if symptoms did not improve within 48 h.  The explanation was short and included the following points: AOM is part of an upper respiratory tract infection, it has been well established that in most cases children will recover regardless of antibiotic prescription, dangerous late complications from AOM unfortunately may occur regardless of whether antibiotics were or were not delivered in the course of the acute illness, and parents were recommended in cases of high fever or severe pain to administer Paracetamol prescribed according to the child’s weight. |
| **Outcomes** | **Primary:**  Antibiotic use  **Secondary:**  Taking antibiotic on first day, association between administration of antibiotics and other variables.   - Antibiotic use was measured by interviewing the parents by phone using a structured questionnaire by a blinded interviewer. |
| **Notes** | - The numbers of events reported were different from the reported percentages.   **Funding:** Not reported. |

| **Poza Abad 2016** | |
| --- | --- |
| **Methods** | RCT, 4 arms.  **Study setting**: Spain, primary care setting. |
| **Participants** | Patients were older than 18 years and had 1 of the following acute, uncomplicated respiratory infections: acute pharyngitis, rhinosinusitis, acute bronchitis, or exacerbation of mild-to-moderate chronic obstructive pulmonary disease (COPD). In all cases, the physician had reasonable doubt as to whether to treat with an antibiotic.  **Number of participants:** 398 |
| **Interventions** | There were three intervention groups:  1) Delayed patient-led prescription strategy (given an antibiotic at first consultation), 2) delayed prescription collection strategy requiring patients to collect their prescription from the primary care reception desk 3 days after the first consultation, and 3) no antibiotic strategy.    Delayed prescription strategies consisted of prescribing an antibiotic to take only if the symptoms worsened or if there was no improvement several days after the medical visit (If they noted no improvement after 5 days (in cases of pharyngitis) or after 10 days (in cases of other infections). |
| **Outcomes** | **Primary:**  Duration and severity of symptoms.  **Secondary:**  Antibiotic use, satisfaction with health care, belief in the effectiveness of antibiotics, absenteeism, risk of complications, risk of need for unscheduled health care.   - Antibiotic use was measured by asking patients by phone or in the follow up visit. |
| **Notes** | **Funding:** The study was sponsored through a governmental grant of the Instituto de Salud Carlos III, Spanish Ministry of Health. One of the authors reported a grant from the Jordi Gol i Gurina Foundation for a research stage at the University of Cardiff in 2013, as well as research grants from the European Commission, Catalan Society of Family Medicine, and Instituto de Salud Carlos III. One of the authors was funded by aMiguel Servet research contract from the Instituto de Salud Carlos. |

| **Taylor 2005** | |
| --- | --- |
| **Methods** | RCT, 2 arms.  **Study setting**: USA, primary care setting. |
| **Participants** | Healthy children younger than 24 months old, seen in the offices of primary care paediatricians. Children and their parents were recruited for the study.  **Number of participants**: 499 |
| **Interventions** | Parental education promoting the judicious use of antibiotics. Parents assigned to the intervention group received a copy of the educational pamphlet, “Your Child and Antibiotics,” developed by the American Academy of Paediatrics, Centers for Disease Control and Prevention and the American Society of Microbiology, and a video in which the main points discussed in the pamphlet were reinforced. The video, which was professionally produced and ran for 5 minutes, featured one of the physicians from the study child’s paediatric practice. Additional copies of “Your Child and Antibiotics,” were mailed to parents of study children at 6 weeks and 6 months after enrolment. |
| **Outcomes** | **Primary:**  Number of diagnoses of otitis media and sinusitis per study child, number of visits per child for which antibiotics (oral or intramuscular) were prescribed for a diagnosis of otitis media, number of visits per child for which antibiotics were prescribed for a diagnosis of otitis media and/or sinusitis and total number of antibiotics prescribed per child.  **Secondary:**  Total number of visits per study patient and number of visits for URI symptoms per child.   - Antibiotic prescription was measured by checking patients’ medical records. |
| **Notes** | **Funding:** The study was supported by GlaxoSmithKline in conjunction with the Aetna Foundation’s Quality Care Research Fund. |

| **Worrall 2010** | |
| --- | --- |
| **Methods** | RCT, 2 arms.  **Study setting**: Canada, primary care setting. |
| **Participants** | Adult patients (aged 18 years or older) with acute upper respiratory tract infections for whom the clinicians thought antibiotic treatment might not be necessary.  **Number of participants:** 149. |
| **Interventions** | Post-dated delayed antibiotic prescription. A delayed prescription dated for 2 days later. The patient was asked to use the prescription only if symptoms had not improved or had worsened after 2 days. |
| **Outcomes** | **Primary:**  Filling the prescription by the patients.  **Secondary:**  The time it took for the patients to fill the prescriptions, the number of early filling (within 2 days), and the reasons given to the pharmacist by patients who filled their delayed prescriptions early.  Filling the prescription was measured by contacting the pharmacies. |
| **Notes** | **Funding:** Not reported. |
